# Supplementary material for: Structure-Based Identification of Novel Histone Deacetylase 4 (HDAC4) Inhibitors
Source: Pharmaceuticals (Basel). 2024 Jul 2;17(7):867. doi: 10.3390/ph17070867 (PMC11279411; doi:10.3390/ph17070867)
Supplement: Supplementary file 1 [file pharmaceuticals-17-00867-s001.zip › pharmaceuticals-3040149-supplementary.pdf]

Structure-based identification of novel histone deacetylase 4 (HDAC4) inhibitors

Rupesh Agarwal[1,2*], Pawat Pattarawat[3], Michael R. Duff[2], Hwa-Chain Robert Wang[3], Jerome Baudry[4], Jeremy C. Smith[1,2*]

1 UT/ORNL Center for Molecular Biophysics, Oak Ridge National Laboratory, TN

2 Department of Biochemistry & Cellular and Molecular Biology, University of Tennessee, Knoxville, TN

3 Department of Biomedical and Diagnostic Sciences, College of Veterinary Medicine, University of Tennessee, Knoxville, TN

4 Department of Biological Sciences, The University of Alabama in Huntsville, Huntsville, Alabama.

*Co-corresponding authors

Supplementary information

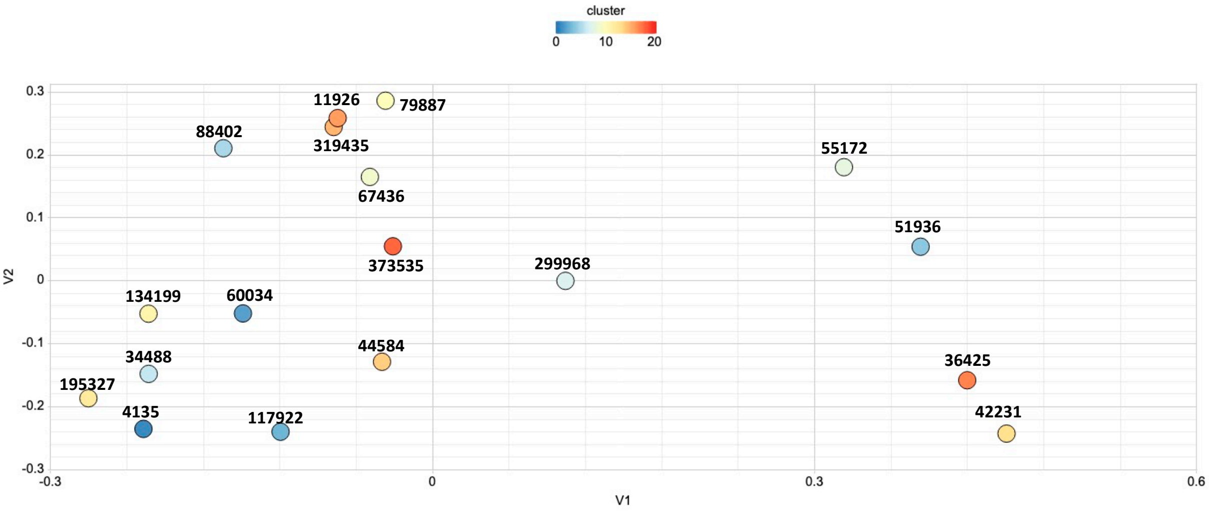

Figure S1: Clustering of compounds by structural and physicochemical similarities by Multidimensional Scaling (MDS) using ChemmineTools[44]. Similarity cutoff of 0.8 is used for coloring only.

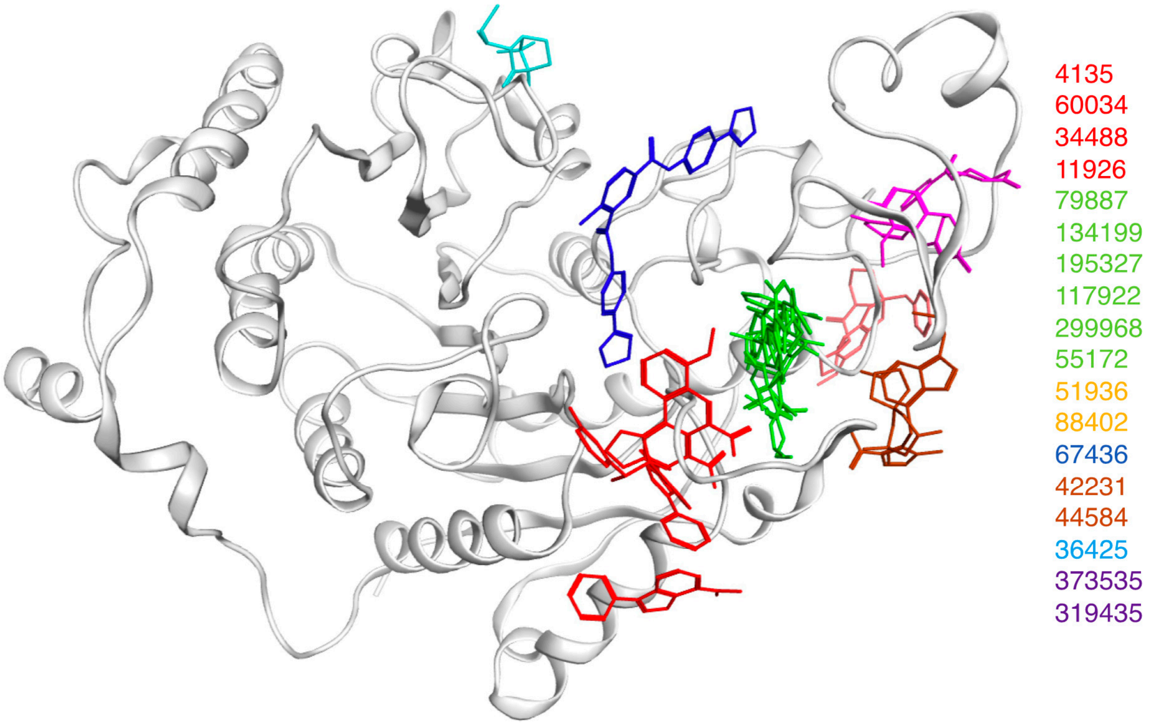

Figure S2: All 18 hit molecules (shown in different colors based on 3D proximity) and their poses on HDAC4 (in the gray ribbon) to show binding site coverage. The figure was made after superimposition of the hits with their respective ensemble structure. All the ensemble structures are not shown for clarity.

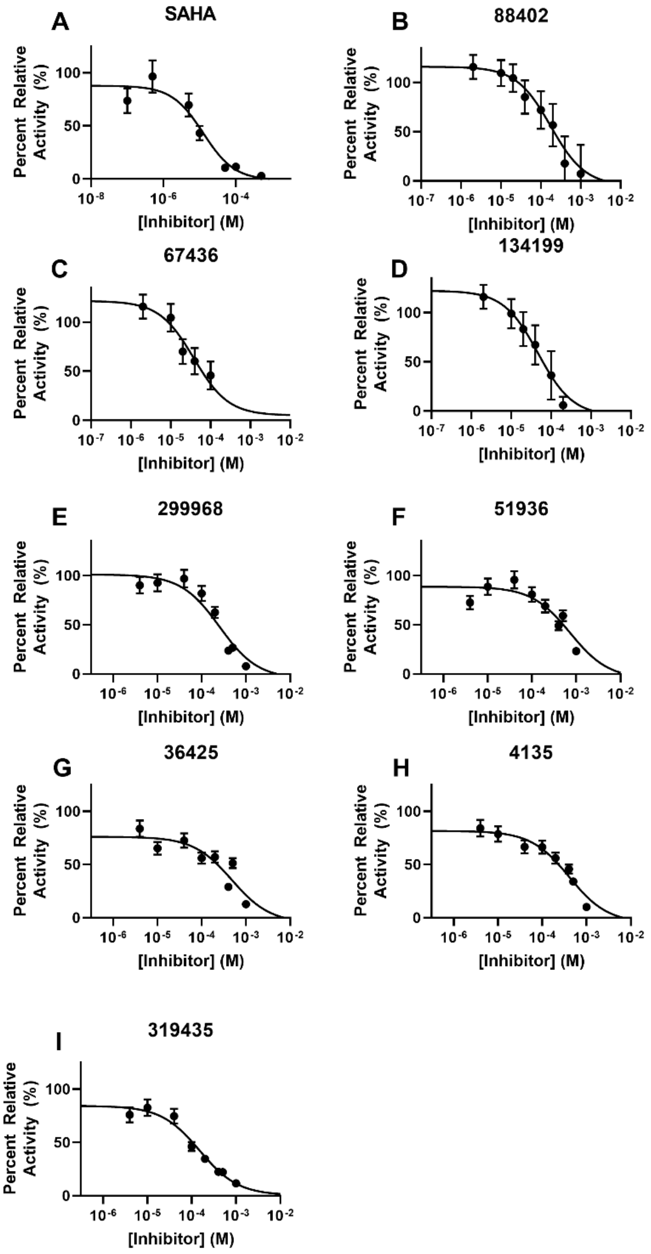

Figure S3. IC50 plots for compounds that inhibit HDAC4.

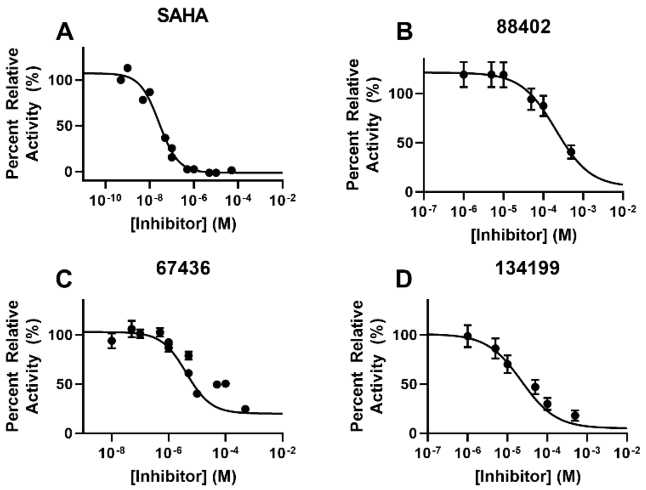

Figure S4. IC50 plots for compounds that inhibit HDAC3.

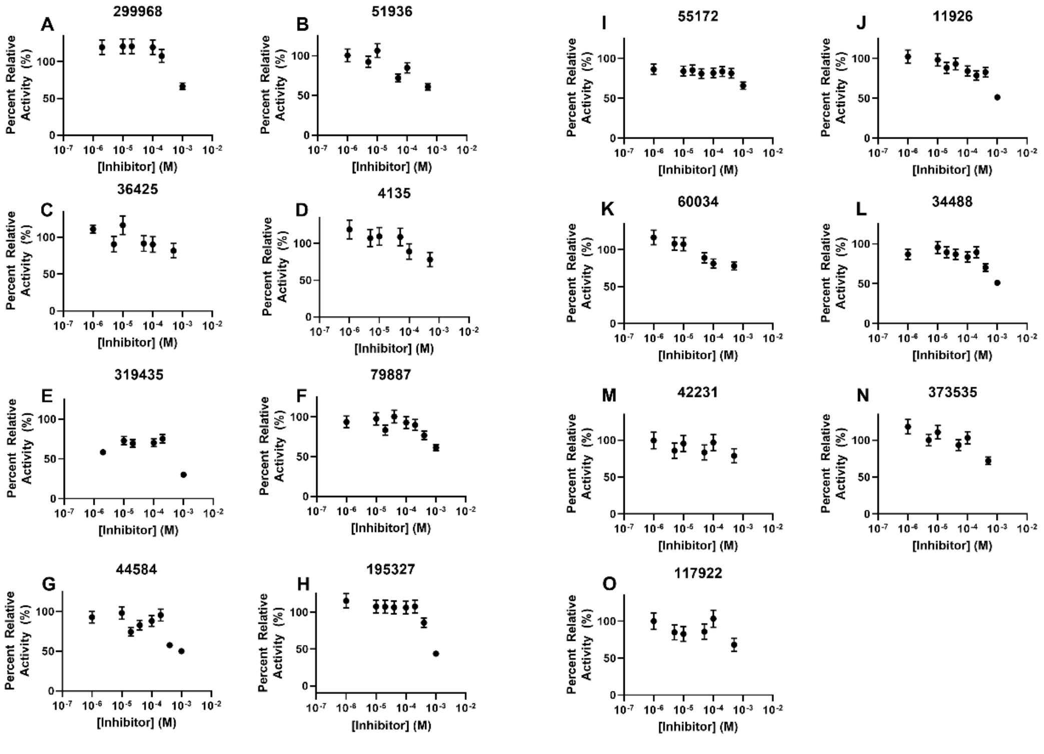

Figure S5. IC50 plots for compounds that yielded no inhibition of HDAC3 below 500 μM.

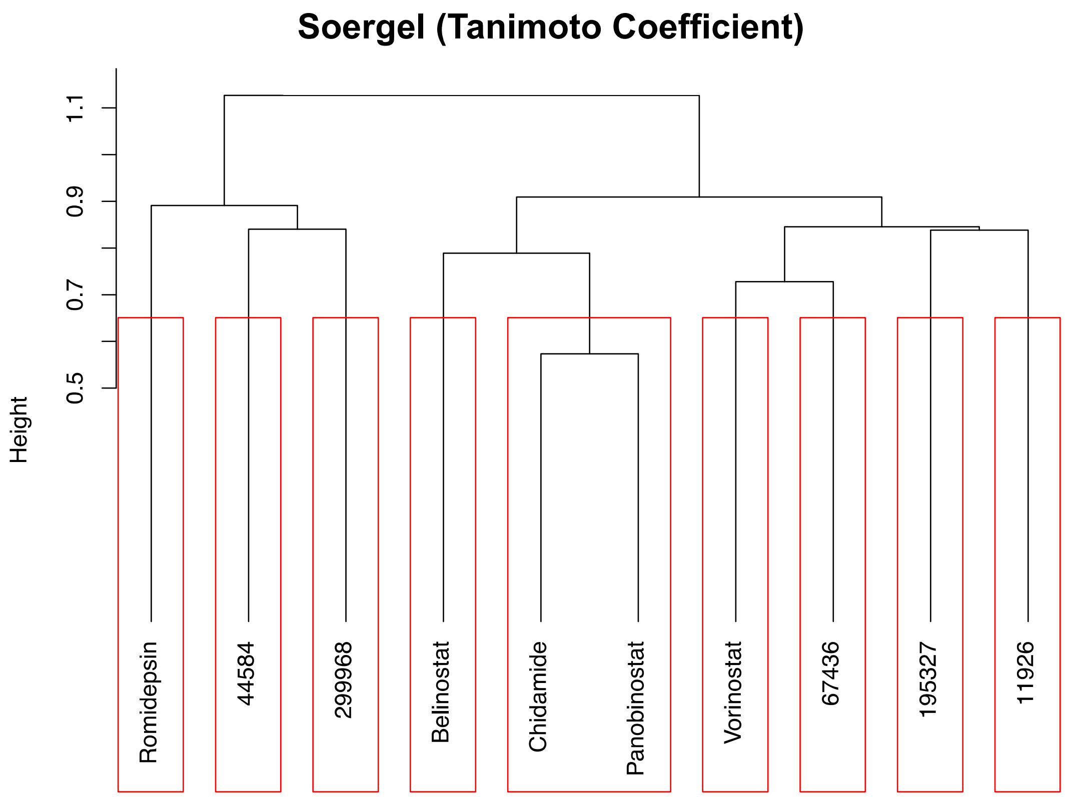

Figure S6: Hierarchical Clustering using MACCS fingerprinting. Distance Method Soergel (Tanimoto Coefficient); Clustering Method: Ward Linkage; Clustering Threshold: 0.7

**Structure**	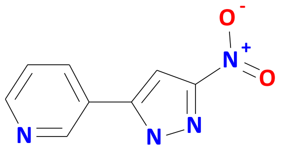	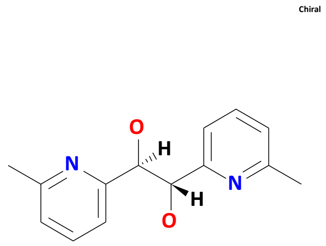	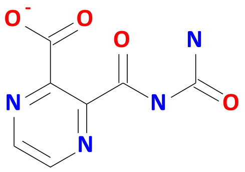
**Name**	**4135**	**60034**	**117922**
**Structure**	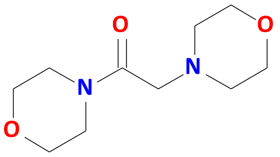	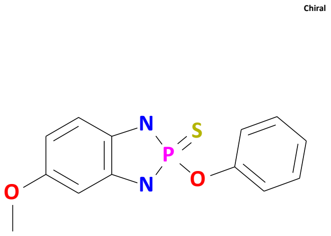	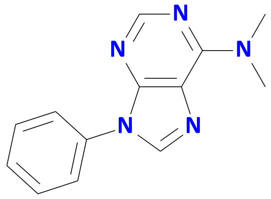
**Name**	**51936**	**88402**	**34488**
**Structure**	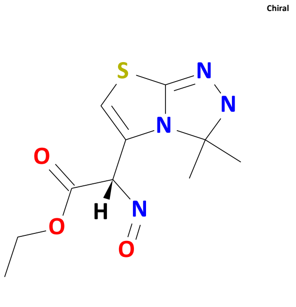	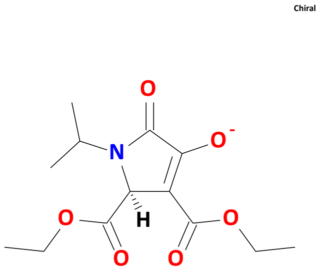	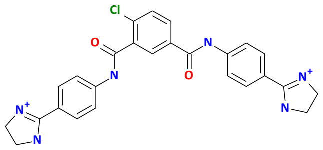
**Name**	**299968**	**55172**	**67436**
**Structure**	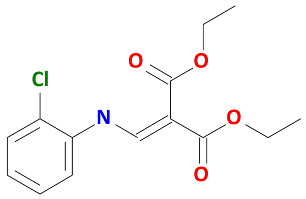	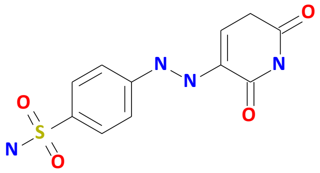	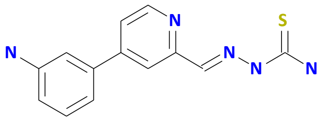
**Name**	**79887**	**134199**	**195327**

**Structure**	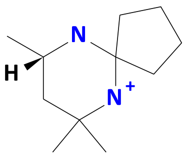	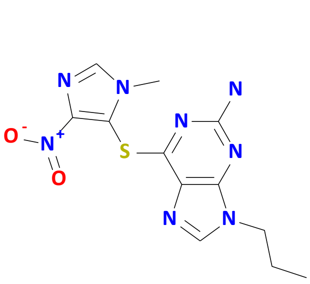	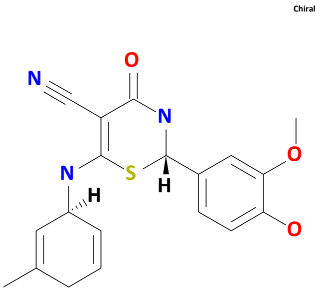
**Name**	**42231**	**44584**	**319435**
**Structure**	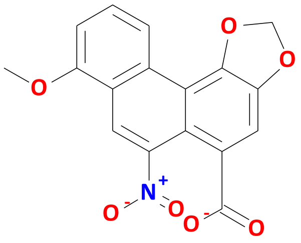	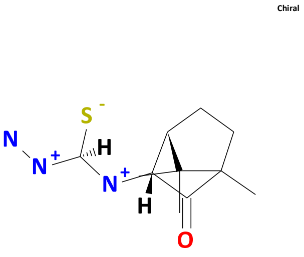	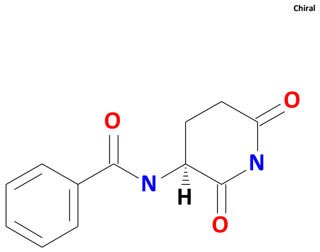
**Name**	**11926**	**36425**	**373535**

Figure S7: Two-dimensional structure of 18 virtual high-scoring compounds.

299968

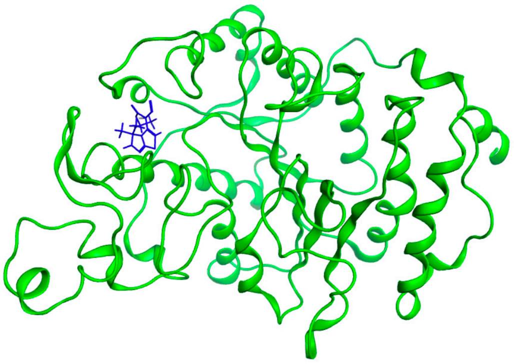

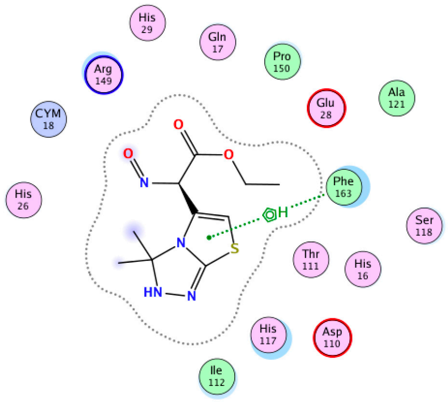

67436

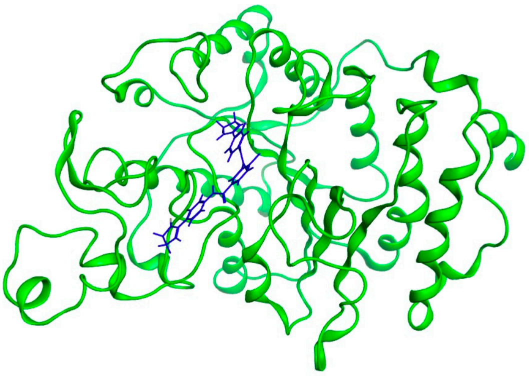

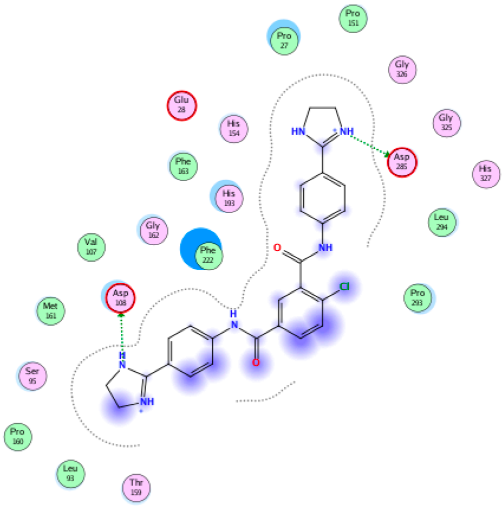

195327

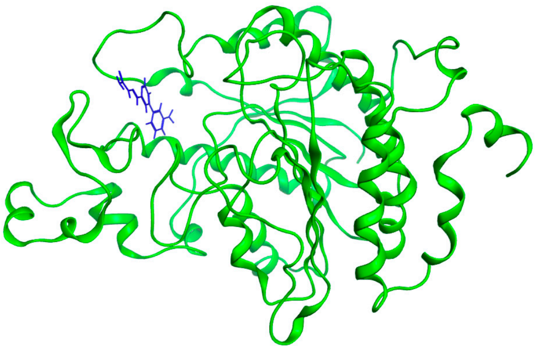

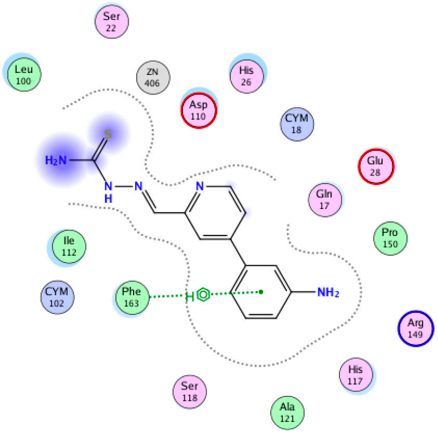

44584

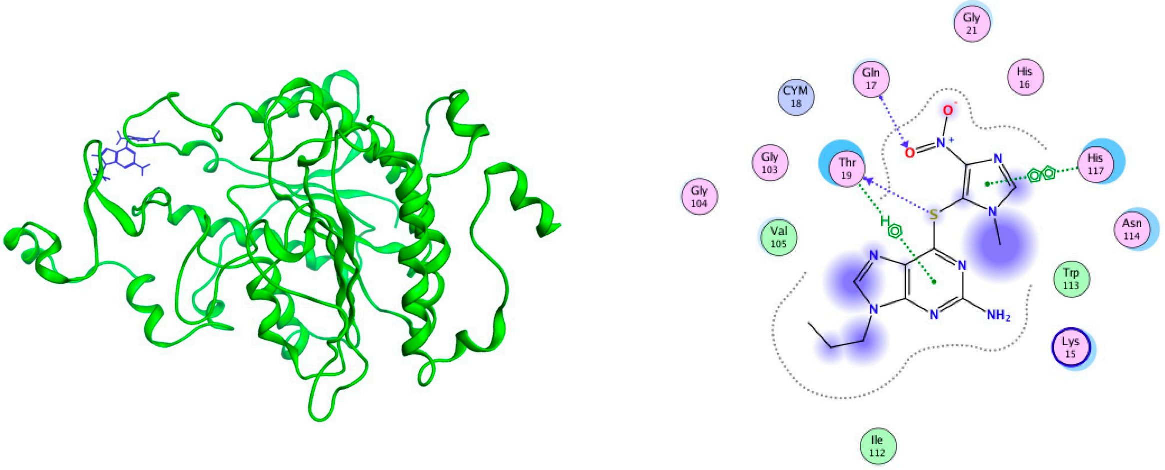

319435

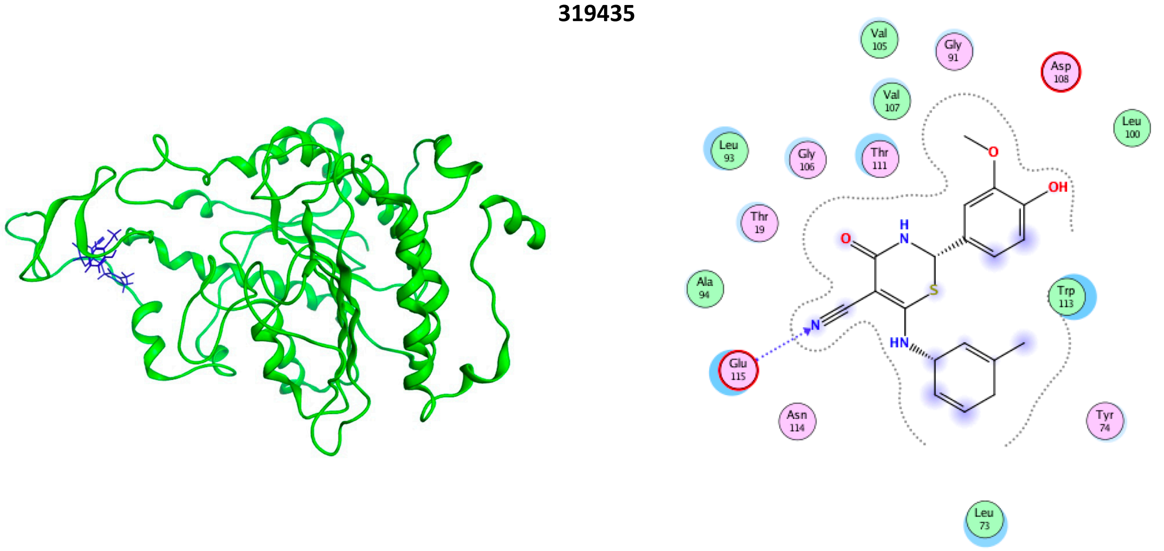

11926

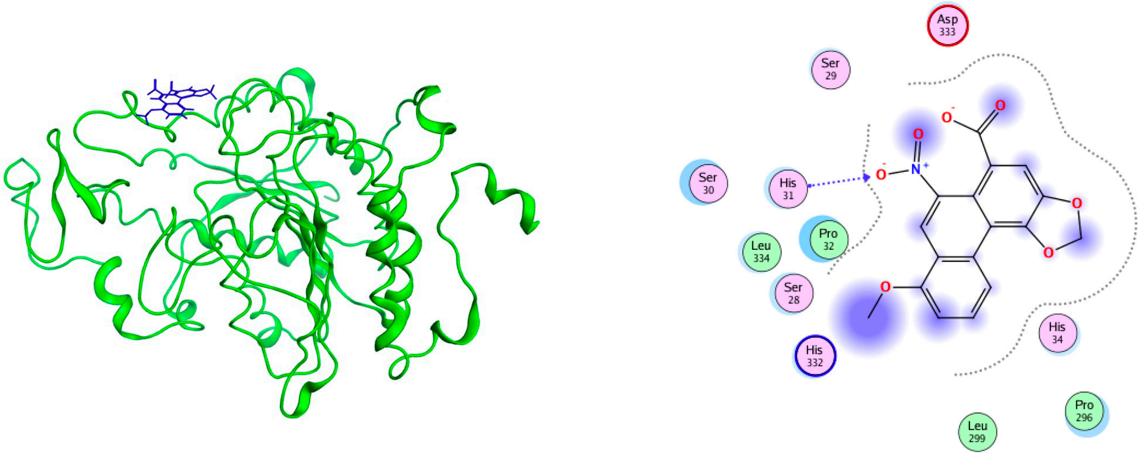

88402

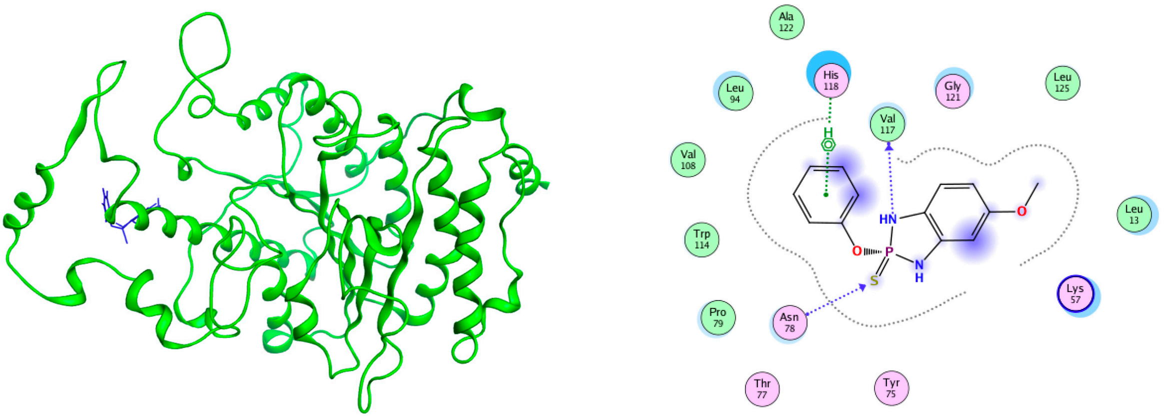

Figure S8: Interaction map of the initial docked pose of the top validated hits in their individual receptor from the ensemble structure.

Table S1: Canonical SMILES of top hits

ID	Canonical SMILES
11926	COC1=CC=CC2=C3C(=C(C=C21)[N+](=O)[O-])C(=CC4=C3OCO4)C(=O)O
44584	CCCN1C=NC2=C1N=C(N=C2SC3=C(N=CN3C)[N+](=O)[O-])N
67436	C1CN=C(N1)C2=CC=C(C=C2)NC(=O)C3=CC(=C(C=C3)Cl)C(=O)NC4=CC=C(C=C4)C5=NCCN5
88402	COC1=CC2=C(C=C1)NP(=S)(N2)OC3=CC=CC=C3
195327	C1=CC(=CC(=C1)N)C2=CC(=NC=C2)C=NNC(=S)N
299968	CCOC(=O)C(=NO)C1=CSC2=NNC(N12)(C)C
319435	CC1=CC(=CC=C1)NC2=C(C(=O)NC(S2)C3=CC(=C(C=C3)O)OC)C#N

Table S2: Binding free energy using the Poisson–Boltzmann surface area (PBSA) method implemented in MOE

ID	GBVI/WSA ΔG (kcal/mol)
11926	-5.5
44584	-5.7
67436	-6.9
88402	-5.7
195327	-5.7
299968	-7.0
319435	-7.3

Table S3: Water-accessible surface area calculated using MOE descriptor (method: a radius of 1.4 A for the water molecule. A polyhedral representation is used for each atom in calculating the surface area.)

ID	Water accessible surface area (Å)	Hydrophobic surface area (Å)	Polar surface area (Å)	IC50 (µM) for HDAC3	IC50 (µM) for HDAC4
11926	512.56238	332.75717	179.80522	>500	ND
44584	538.22272	324.91504	213.30766	>500	ND
67436	809.40997	571.96582	237.44415	9.2 ± 5.2	47±28
88402	501.51611	373.22766	128.28844	210 ± 60	100±40
195327	519.33081	247.93991	271.3909	>500	ND
299968	439.53036	232.28741	207.24297	>500	250 ± 170
319435	570.80597	321.4003	249.40564	>500	150 ± 50
